# Dietary chokeberry and dried jujube fruit attenuates high-fat and high-fructose diet-induced dyslipidemia and insulin resistance via activation of the IRS-1/PI3K/Akt pathway in C57BL/6 J mice

**DOI:** 10.1186/s12986-019-0364-5

**Published:** 2019-06-03

**Authors:** Oeuk Jeong, Hyun-Sook Kim

**Affiliations:** Department of Food and Nutrition, College of Human Ecology, Sookmyung Women’s University, Sunhun building 307, Cheongpa-ro 47-gil 100 (Cheongpa-dong 2(i)-ga), Yongsan-gu, Seoul, 04310 South Korea

**Keywords:** Metabolic syndrome, Insulin resistance, Dyslipidemia, Glucose metabolism, Lipid metabolism, Chokeberry, Dried jujube fruits

## Abstract

**Background:**

The incidence of metabolic syndrome linked to dyslipidemia and insulin resistance has increased; thus, studies must be conducted to elucidate this phenomenon. The present study aimed to investigate the protective effects of chokeberry and dried jujube diet on high-fat and high-fructose diet-induced dyslipidemia in mice.

**Methods:**

Male C57BL/6 J mice were divided into five groups: ND, mice fed normal diet and tap water; HFFD, mice fed 60% high-fat and 10% fructose diet (HFFD) in tap water; HFFD+C, mice fed HFFD with 1% chokeberry powder; HFFD+J, mice fed HFFD with 1% jujube fruit powder; and HFFD+M, mice fed HFFD with 0.5% chokeberry + 0.5% jujube fruit powder mixture.

**Results:**

After 10 weeks of dietary treatment, chokeberry and dried jujube fruits reduced HFFD-induced weight gain and central obesity and decreased liver weight and abdominal and epididymal fat mass. Furthermore, such fruits attenuated HFFD-induced dyslipidemia; decreased triglyceride, total cholesterol, non-high-density lipoprotein-cholesterol, low-density lipoprotein-cholesterol, and very-low-density lipoprotein-cholesterol levels. Insulin resistance was improved via the consumption of dietary chokeberry and dried jujube fruits according to various indicators (serum insulin level, fasting blood glucose level, homeostatic model assessment-insulin resistance score, and oral glucose tolerance test value). These treatments were found to lower serum triglyceride levels. Moreover, the consumption of chokeberry and dried jujube changed the hepatic protein expression of insulin receptor, insulin receptor substrate 1, phosphoinositide 3-kinase, Akt, and catalase, which are associated with insulin resistance.

**Conclusions:**

Chokeberry and dried jujube could be used in the management of dyslipidemia and insulin resistance associated with metabolic syndrome by reducing risk parameters in mice with HFFD.

## Background

High-fat and high-fructose diet (HFFD), which is a western diet, is generally composed of carbohydrates, such as fructose and sucrose, as well as saturated fat. This diet has been associated with numerous diet-induced complications, such as obesity, type II diabetes mellitus (T2DM), and metabolic syndrome (MetS), which is a pathological state characterized by hypertension, abdominal obesity, dyslipidemia, and hyperglycemia, and such characteristics are considered major public health problems worldwide. Their complex conditions are significantly correlated to overweight or obesity and insulin resistance, which is an important factor in the development of MetS and T2DM. In particular, hepatic insulin resistance is correlated to a decrease in insulin signal transmission for inhibiting glucose production and insulin-stimulated hepatic lipogenesis [1]. In the Republic of Korea, the prevalence of MetS in adults over 30 is reported to be 30% according to a report from Korea Centers for Disease Control and Prevention (KCDC). For this reason, there are continuing needs to prevention study to relieve the MetS in South Korea.

Various studies have been shown that polyphenol-rich diet could reduce the risk of developing MetS [2–6]. Polyphenols have potent antioxidant properties and perform a variety of biological functions such as anticancer and anti-inflammatory properties, and lipid homeostasis [2, 7–9]. For this reason, the relationship between polyphenols in fruits and various health problems is being assessed in research studies. According to the previous studies, polyphenols containing flavonoids could contribute the health benefits by improvement of insulin receptor substrate (IRS)/ phosphoinositide 3-kinase (PI3K)/Akt pathway [10] and of insulin sensitivity with reduced markers of inflammation [11]. The association between the HFFD diet and the IRS/PI3K/Akt pathway will be described in the following paragraphs.

Several studies have investigated the actual mechanism of MetS and insulin receptor (IR)/IRS1 with PI3K/Akt pathway, which is a downstream of the insulin signaling pathway. Insulin receptor, a glycoprotein consisting of an extracellular α-subunit (135 kDa) and a transmembrane β-subunit (95 kDa), is an allosteric enzyme in which the α-subunit inhibits the tyrosine kinase activity of the β-subunit [12]. IR combined with insulin initiates a cascade of phosphorylation events, including that of downstream as well as the IRS and PI3K/Akt pathway [13, 14]. Activation of IRS/PI3K/Akt pathway is the main target that alleviating dyslipidemia, insulin resistance and obesity [12, 15–17].

Insulin is secreted by the β-cells of the pancreatic islet of Langerhans. The secreted insulin binds to the IR that traverses the cell membrane. Consequently, they transmit insulin signaling across the cell membrane, resulting in a sequence of further phosphorylation reactions, such as that observed for PI3K [14]. Akt, which is also called protein kinase B, is a key downstream effector of the PI3K pathway. Akt mediates most of the metabolic effects of insulin, thereby regulating glucose transport, lipid synthesis, gluconeogenesis, and glycogen synthesis. Moreover, it plays a role in the control of cell cycle and survival [13, 18]. The activation of PI3K generated phosphatidylinositol-3,4,5-triphosphate (PIP3) from phosphatidylinositol-4,5-bisphosphate (PIP2), and as a result, insulin affects metabolism by activating Akt via phosphorylation (at T^308^ and S^473^) [12]. Activated Akt promotes the downstream effectors, which regulates glucose metabolism via FoxO protein, particularly FoxO1 and glucose 6-phosphate and glycogen synthase kinase 3 (GSK-3) and lipid metabolism via mTOR complex 1 (mTORC1) and sterol regulatory element-binding proteins (SREBP) [19]. Therefore, activation of IRS/PI3K/Akt pathway modulates the risk of MetS including dyslipidemia and insulin resistance.

Black chokeberry (*Aronia melanocarpa*) is the main breed of the *Aronia melanocarpa* species, and it is classified according to color difference between red and black. Chokeberry is a member of the Rosaceae family, and it contains several phytochemicals, such as total polyphenols, procyanidins, anthocyanins, and flavonols [20, 21]. Moreover, it is known as a super food worldwide and has been used as a research material to elucidate the effects of dietary intake. Such fruits have been extensively used to validate protective effects against aging [22], dyslipidemia [23], hyperglycemic state [24, 25], liver damage [23, 26, 27], and hypertension [5]. Chokeberry has been used in limited industrial productions due to its strong sour taste, bitterness, and unripe smell. In relation to this reason, such fruit is blended with foods to compensate for its undesirable taste and smell rather than being used alone [20].

Jujube (*Ziziphus jujuba*) is considered a health-promoting food in Asia. Such fruit is dried to increase its nutrient contents, and it has been used as a traditional medicine in Asia. According to previous studies, the nutritional contents of jujube could change during drying process [28, 29]. In a review article, the jujube fruit contains minerals, vitamins, polyphenols, flavonoids, anthocyanins, and proanthocyanidins [30]. Dried jujube has beneficial effects on hepatoprotection [31], diabetes [32], dyslipidemia [33], and inflammation [34].

As interest in the consumption of health-promoted food containing fruits increased in Korea, research was needed to establish scientific evidences of protective effect. Therefore, the current study was conducted whether the effects of chokeberry and jujube fruits could reduce the risk of MetS when consumed individually or in combination. Many studies reported chokeberry consumption led to reduce the risk of obesity, however, it is difficult to applicate the individual consumption because of bitterness. Nowadays, functional foods in combination form are being developed. Despite these efforts, the scientific evidences are insufficient. The tests on availability continue, however there are weaknesses. This is because most tests are only been conducting on the antioxidative activities of food without confirming changes of the specific mechanism, which promotes health. This study is the only experiment that directly feeding in form of fruit powder to mice with MetS induced by HFFD diet and have specialty because the present study conducted health-promoting effects with combination form of different fruits. Therefore, there is uniqueness and originality in current study.

This study aimed to investigate the effects of dietary chokeberry and/or dried jujube fruits on central obesity, lipid homeostasis, glucose intolerance, and insulin resistance that are induced by HFFD in C57BL/6j mice by controlling the IRS/PI3K/Akt pathway. These data would provide sound scientific evidence for the clinical treatment of dyslipidemia and insulin resistance.

## Methods

### Ethical approval

This experimental design was approved by the Institutional Animal Care and Use Committee (IACUC) of Sookmyung Women’s University (SMWU-IACUC-1701-044).

### Experimental scheme

Lipid and glucose metabolism have been documented as important risk factors of metabolic disorder. Thus, this study aimed to investigate the synergistic effects of chokeberry (*Aronia melanocarpa*) and jujube (*Ziziphus jujuba* Mill.) diet on lipid metabolism and antioxidant capacity in HFFD-induced metabolic disorders in the C57BL/6 J mice model.

**Fig. 1:**
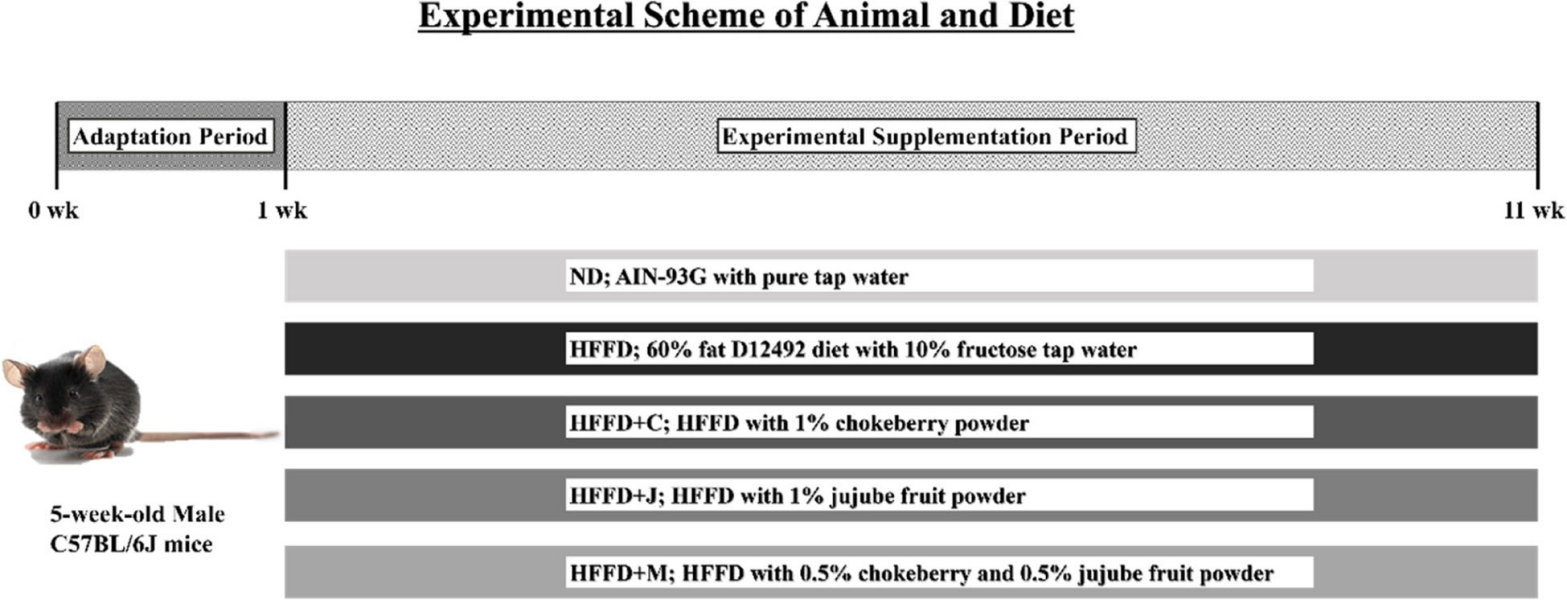
Experimental Scheme of Animal and Diet. In the current study, 6-week-old C57BL/6 J mice were divided into five groups; ND (normal diet with tap water), HFFD (60% of fat with 10% of fructose water), HFFD+C (HFFD with 1% of chokeberry powder), HFFD+J (HFFD with 1% jujube fruit powder), and HFFD+M (HFFD with mixture that contains 0.5% chokeberry and 0.5% jujube fruit powder)

After the acclimation period, 6-week-old C57BL/6 J mice were divided into five groups: ND, mice fed with normal diet and tap water; HFFD, mice fed with HFFD (60% of fat, 10% of fructose) in tap water; HFFD+C, mice fed with HFFD with 1% chokeberry powder and 10% fructose in tap water; HFFD+J, mice fed with HFFD with 1% jujube fruit powder and 10% fructose in tap water; and HFFD+M, mice fed with HFFD with 0.5% chokeberry + 0.5% jujube fruit powder mixture and 10% fructose in tap water (Fig. 1).

The animals were euthanized with CO_2_ after 10 weeks of treatment. Their serum, organs (heart, kidneys, lung, and liver), and tissues (abdominal fat and epididymal fat) were isolated. Serum was separated by centrifugation at 3000 rpm for 45 min and stored at − 70 °C until analysis. For liver analysis, the liver samples were homogenized, and hepatic triglyceride (TG) level was measured. Moreover, hepatic protein expression of Insulin Receptor (IR), Insulin Receptor Substrate 1 (IRS-1), PI3K, phosphor-PI3K (p-PI3K), Akt, phosphor-Akt (p-Akt), and Catalase (CAT) were measured via Western blot analysis.

#### Animals and diets

The 5-week-old male C57BL/6 J mice (Saeronbio Inc., Gyeonggi-do, Korea) were housed with controlled temperature (21 ± 1 °C) and humidity (50–60%) conditions in a 12-h light/dark cycle throughout the study. They had free access to water and chow diet.

After 1 week of acclimation, the 6-week-old mice were randomly divided into five groups (*n* = 7 for each group): [1] ND, mice fed with normal diet (AIN-93G) and tap water; [2] HFFD, mice fed with HFFD (60% fat, 10% fructose) in tap water; [3] HFFD+C, mice fed with HFFD with 1% chokeberry powder and 10% fructose in tap water; [4] HFFD+J, mice fed with HFFD with 1% jujube fruit powder and 10% fructose in tap water; and [5] HFFD+M, mice fed with HFFD with 0.5% chokeberry and 0.5% jujube fruit powder mixture and 10% fructose in tap water. The normal diet was the AIN-93G diet (Research diet, New Brunswick, NJ, the USA), and the high-fat diet was the D12492 diet (Research diet, New Brunswick, NJ, the USA). The experimental scheme of the current study is shown in Fig. 1.

Fresh chokeberries were purchased from Ofresh (Gochang, Jeonbuk, Korea) and freeze dried for 2 days (Bondiro MCFD 8508 Freeze Dryer, Ilshin, Seoul, Korea). Dried jujubes were purchased from Boeun Dule farm (Boeun, Chungbuk, Korea). Freeze-dried chokeberry and dried jujube were grounded and mixed to the diet. All diets, except the normal diet, were isocaloric. The nutritional characterization of chokeberry and jujube fruit powders are listed in Table 1. Table 2 shows the composition of each experimental diets. The percentage of chokeberry (1%) was chosen according to the previous pilot studies which investigated by our laboratory [22, 26, 35]. We decided and tested the 1% of jujube fruits as the same dose with chokeberry. Moreover, we chose to use 0.5% of chokeberry and 0.5% of dried jujube fruits (HFFD+M) in the mixed powder group because we hoped to match that experimental groups were equal to 1% of the total intake.

**Table 1:** Nutritional components of chokeberry and jujube fruits

	Chokeberry	Jujube fruits
Calories (kcal/100 g)	306	321
Moisture(g/100 g)	6.8	11.4
Ash(g/100 g)	3.6	2.6
Carbohydrate(g/100 g)	81.5	79.0
Protein(g/100 g)	6.0	6.6
Fat(g/100 g)	2.1	2.6
Glucose(g/100 g)	4.2	8.8
Fructose(g/100 g)	5.0	9.7
Sucrose(g/100 g)	0.1	8.3
Dietary fiber(g/100 g)	31.5	12.6

The result of major nutritional composition of 2 samples was tested by Korea Food Research Institute (Gyeonggi-do, Republic of Korea), and all value is mean

**Table 2:** Composition of experimental diets

	Experimental diet				
	ND	HFFD	HFFD + C	HFFD + J	HFFD + M
Macronutrient composition					
Carbohydrate, % of energy	63.90	20.00	20.65	20.61	20.64
Protein, % of energy	20.30	20.00	19.86	19.87	19.87
Fat, % of energy	15.80	60.00	59.49	59.52	59.50
Energy, kcal/kg	4000.00	4057.00	4047.03	4048.53	4047.78
Ingredients (g/kg)					
Casein, 30 Mesh	200.00	258.45	255.87	255.87	255.87
L-cystine	3.00	3.88	3.84	3.84	3.84
Corn starch	397.00	0.00	0.00	0.00	0.00
Maltodextrin 10	132.00	161.53	159.91	159.91	159.91
Sucrose	100.00	88.91	88.02	88.02	88.02
Cellulose, BW200	50.00	64.61	63.96	63.96	63.96
Soybean Oil	70.00	32.31	31.99	31.99	31.99
Lard	–	316.60	313.43	313.43	313.43
Mineral Mix S10022G	35.00	–	–	–	–
Mineral Mix S10026	–	12.92	12.79	12.79	12.79
DiCalcium Phosphate	–	16.80	16.63	16.63	16.63
Calcium Carbonate	–	7.11	7.04	7.04	7.04
Potassium Citrate, 1 H2O	–	21.32	21.11	21.11	21.11
Vitamin Mix V10037	10.00	–	–	–	–
Vitamin Mix V10001	–	12.92	12.79	12.79	12.79
Choline Bitartrate	2.50	2.58	2.55	2.55	2.55
Chokeberry powder	–	–	10.00	–	5.00
Jujube fruit powder	–	–	–	10.00	5.00
Total (g)	999.50	999.94	999.94	999.94	999.94

^a^ AIN-93G as control diet for ND group

^b^ 60% High fat diet with 10% fructose drinking water (western diet) for HFFD group

*ND*: Normal diet group, *HFFD*: High fat and high fructose (HFFD) diet group, HFFD+C: HFFD with 1% chokeberry powder group, HFFD+J: HFFD with 1% jujube fruits powder group, HFFD+M: HFFD with 0.5% chokeberry + 0.5% jujube fruits mixed powders group

#### Measurement of body weight, food intake, and water intake

Body weight for each animal was recorded weekly. Food intake and water intake were measured every 2 days, and body weight was evaluated once a week during the feeding period. The animals were weighed weekly, starting from the day of arrival. The final body weight was measured a day before the sacrifice. The food efficiency ratio (FER) was calculated using the following equation:

FER = total body weight gain (g) / total food intake (g) × 100.

#### Blood and tissue preparation

After an overnight fast, the final body weight was measured, and the mice were euthanized using CO_2_. Blood sample was collected via cardiac puncture to determine the serum lipid profiles. Serum was separated by centrifugation at 3000 rpm for 45 min (Combi-450R, Hanil Co. Ltd., Seoul, Korea) and stored at − 70 °C until analysis (DF8517; Ilshin Laboratory Co., Ltd., Seoul, Korea). Hearts, kidneys, lungs, livers, abdominal fats, and epididymal fats were isolated and measured with an electronic balance (OHAUS, NJ, the USA). All organs were stored at − 70 °C until analysis (DF8517; Ilshin Laboratory Co., Ltd., Seoul, Korea). The organ coefficient of each organ was calculated with following equation:

$$ \mathrm{organ}\ \mathrm{coefficient}\ \left(\mathrm{g}/100\ \mathrm{g}\right)=\mathrm{organ}\ \mathrm{weight}\ \left(\mathrm{g}\right)/\mathrm{body}\ \mathrm{weight}\ \left(\mathrm{g}\right)\times 100 $$

#### Glucose metabolism profiles

1. Fasting blood glucose level, serum insulin concentration, and HOMA-IR score

Fasting glucose concentration was directly evaluated using the GlucoCard X-Meter (Arkray, Kyoto, Japan). Serum insulin levels were measured using the Insulin Mouse ELISA kit (80-INSMS-E01, ALPCO, Salem, NH). The Homeostatic Model Assessment-Insulin Resistance (HOMA-IR) score, which is called the homeostasis model assessment of insulin resistance, was calculated using fasting blood glucose and insulin concentrations.

$$ \mathrm{HOMA}-\mathrm{IR}\ \mathrm{score}=\mathrm{fasting}\ \mathrm{blood}\ \mathrm{glucose}\ \left(\mathrm{mmol}/\mathrm{L}\right)\times \mathrm{serum}\ \mathrm{insulin}\ \left(\mathrm{pmol}/\mathrm{L}\right)/22.5 $$

2. Oral glucose tolerance tests

After 10 weeks of consuming the experimental diet, according to their respective groups, oral glucose tolerance tests (OGTTs) were performed. All mice were fasted overnight and then provided with 2 g/kg d-glucose solution. Whole blood was collected from the caudal vein, and blood sugar level was measured using a glucose monitoring device (Arkray, Kyoto, Japan) immediately after treatment at 0, 30, 60, 90, and 120 min.

#### Lipid profiles

1. Serum TG, TC, HDL, LDL, and VLDL levels

Serum TG and total cholesterol (TC) levels were measured with the TG-S kit (3I1570, Asanpharm, Hwaseong, Korea) and T-CHO kit (3I2020, Asanpharm, Hwaseong, Korea), respectively. The HDL-CHO kit (3I2030, Asanpharm, Hwaseong, Korea) was used to measure serum high-density lipoprotein cholesterol (HDL-C) levels.

Low-density lipoprotein and very-low-density lipoprotein cholesterol (LDL-C and VLDL-C) levels were calculated using the Friedewald equation [36] as follows:

LDL-C level (mg/dL) = TC level-(HDL-C level + TG level/5) (mg/dL)

VLDL-C level (mg/dL) = TG level/5 (mg/dL)

2. Atherosclerotic index (AI) and cardiac risk factor (CRF)

Atherosclerotic index (AI) and cardiac risk factor (CRF) were calculated using the following formula [36, 37]:

AI = (TC level – HDL-C level)/HDL-C level

CRF = TC level/HDL-C level

#### Liver TG levels

Total liver lipid levels were obtained using the Folch method [38]; then, liver TG levels were determined. Briefly, the liver samples were homogenized with chloroform/methanol (2:1) solution to a final dilution of 20 folds than the volume of the tissue sample. The samples were mixed for 15 min and were centrifuged at 1000 rpm for 5 min at room temperature. The upper phase was removed as completely as possible with a pipette. Next, the lower phase was evaporated on a hot plate until chloroform disappeared. Liver TG levels were identified using the TG-S kit (3I1570, Asanpharm, Hwaseong, Korea) and were measured.

#### Western blot analysis

In total, 8 mg of liver tissue was used to extract protein in the liver with the Pro-prep kit (17,081, iNtRON biotechnology, Gyeonggi-do, Korea). Protein concentration was measured using the PRO-MEASURE™ kit (21,011, iNtRON biotechnology, Gyeonggi-do, Korea). Protein samples were resolved on the SDS-PAGE and were transferred to the polyvinylidene difluoride (PVDF) membrane (Merck Millipore, MA, the USA) via electrophoretic transfer (Bio-Rad Laboratories, Inc., Hercules, CA, the USA). The membrane was pre-blocked in 5% skim milk containing phosphate-buffered saline solution and 0.1% tween-20 (PBST) for an hour. Then, the membrane was incubated overnight at 4 °C with the following primary antibodies: Insulin Receptor (IR, 1:1000, Abcam, Cambridge, U.K.), Insulin Receptor Substrate 1 (IRS-1, 1:1000, Cell Signaling Technology Inc., MA, USA), PI3 kinase class III antibody (PI3K, 1:500, Cell Signaling Technology Inc., MA, USA), PI3 kinase p85 alpha + gamma (Tyr467 + Tyr199) antibody (p-PI3K, 1:500, Bioss, MA, USA), Akt polyclonal antibody (Akt, 1:1800, Abnova, Taipei, Taiwan), phospho-Akt (Ser 473), antibody (p-Akt, 1:1000, Cell Signaling Technology, Inc., MA, the USA), CAT (1:1000, Abnova, Taipei, Taiwan). After incubation, the membrane was washed with PBST for 10 min for 3 times in a row. Then, the membrane was incubated in goat anti-rabbit IgG H&L (HRP) secondary antibody (1:7500, Abnova, Taipei, Taiwan) and donkey anti-goat secondary antibody (1:7500, Abnova, Taipei, Taiwan). Protein level was normalized via glyceraldehyde 3-phosphate dehydrogenase (GAPDH) expression with GAPDH polyclonal antibody (1:1000, Abnova, Taipei, Taiwan). Immobilon Western horseradish peroxidase substrate (Merck Millipore, Bedford, MA, the USA) was used for chemiluminescence detection. The immunoreactive band intensities were quantified via densitometric analysis (LAS-3000, Fujifilm Co., Tokyo, Japan).

#### Statistical analysis

Statistical analysis was performed with SAS 9.4 (SAS Institute Inc., Cary, NC, the USA). All data were presented as mean ± SD. The results for each experimental group were compared using one-way analysis of variance (ANOVA). Differences in mean values between the five groups were tested with the Duncan’s multiple tests. A *p* value <.05 was considered statistically significant.

## Results

### Body weight, food intake, and food efficiency ratios

**Fig. 2:**
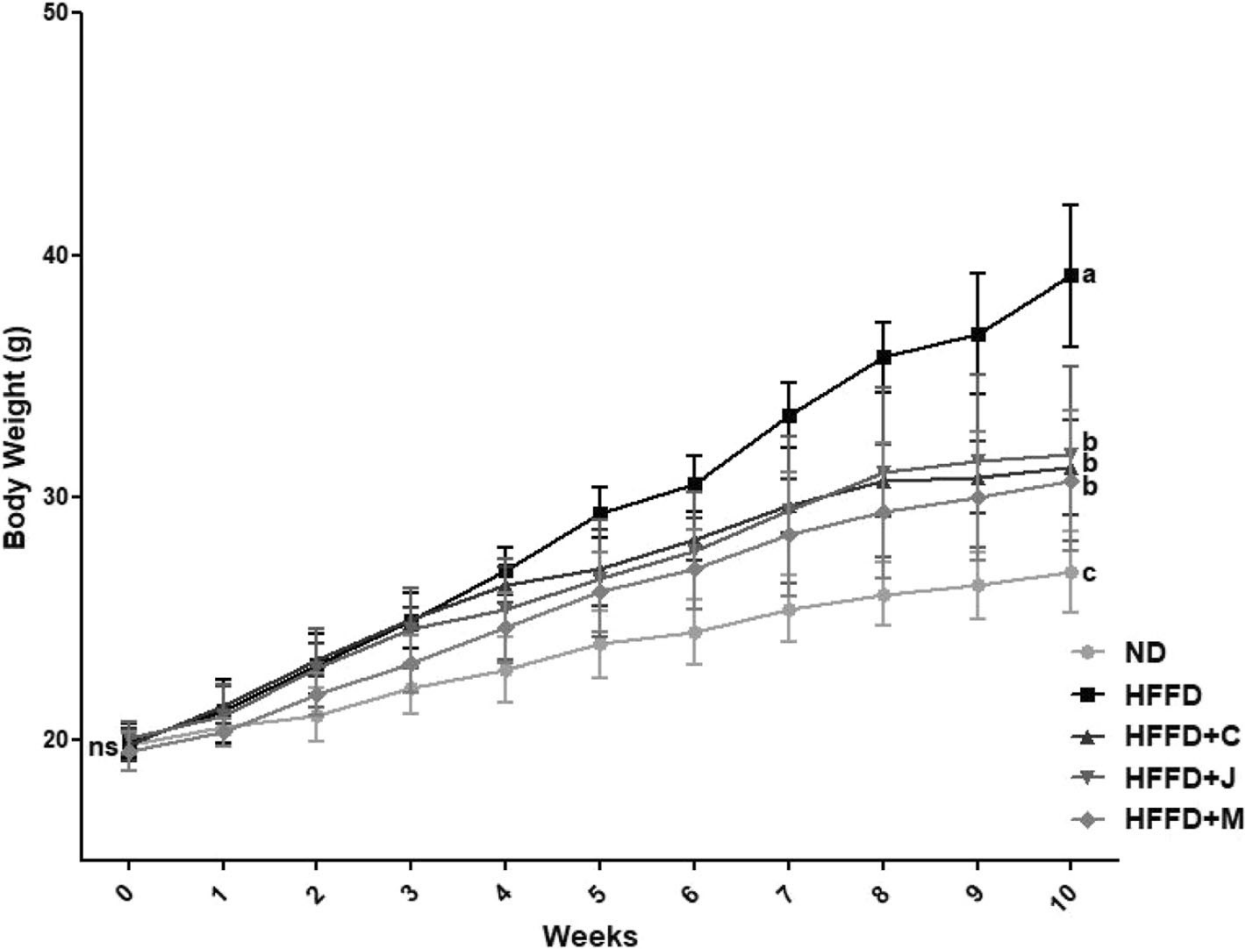
Body Weight Changes of Each Group. Values are means **±** SD (*n* = 7). The different letters (a, b, c) within a column indicate significant difference (*p* < .05) determined by Duncan multiple range test. Abbreviation: ns, not significant. ND: normal diet group, HFFD: high fat and high fructose (HFFD) diet group, HFFD+C: HFFD with 1% chokeberry powder group, HFFD+J: HFFD with 1% jujube fruits powder group, HFFD+M: HFFD with 0.5% chokeberry + 0.5% jujube fruits mixed powders group

Body weight changes are presented in Fig. 2. At the start of the experiment period, body weight was not significantly different among all five groups. A significant elevation in the final body weight and body weight gain was observed in the group with high-fat and high-fructose diet for 10 weeks compared with the group with normal diet (*p* < .000 l). Compared with the HFFD group, the final body weight and body weight gain were reduced in the three groups fed with three fruits (*p* < .0001 for all groups). According to consumption of chokeberry and/or dried jujube fruits, there was significant decreases in HFFD+C, HFFD+J, and HFFD+M group in comparison with HFFD group although it was not up to the level of ND group. The weight changes in HFFD+C, HFFD+J and HFFD+M group were reduced by 31.42, 27.66 and 15.94% respectively, relative to the weight changes of the HFFD group.

Calorie intake per day did not significantly differ in all groups (*p* = 0.0865). The FER (%) of the HFFD group was statistically higher than the ND group (*p* < .0001). The HFFD+C, HFFD+J, and HFFD+M groups had a significantly lower FER than the HFFD group (*p* < .0001). Results of all groups are as follows: ND group with 4.03 ± 0.83%, HFFD group with 10.15 ± 2.62%, HFFD+C group with 7.70 ± 1.58%, HFFD+J group with 8.36 ± 1.91%, and HFFD+M group with 8.67 ± 1.91%. **(**Table 3**).**

**Table 3:** Food intake and food efficiency ratio of each group

	Food intake (g/day)	FER
Controls		
ND	2.66 ± 0.07^a^	4.03 ± 0.83^c^
HFFD	2.24 ± 0.14^b^	10.15 ± 2.62^a^
Experiments		
HFFD+C	2.04 ± 0.14^c^	7.70 ± 1.58^b^
HFFD+J	1.97 ± 0.10^c^	8.36 ± 1.91^b^
HFFD+M	2.23 ± 0.22^b^	8.67 ± 1.91^b^

Values are expressed as mean ± S.D. Different letters (a > b > c) within a column indicate significant differences (*p* < .05) determined by Duncan’s multiple range test. FER, food efficiency ratio; ND: normal diet group, HFFD: high fat and high fructose (HFFD) diet group, HFFD+C: HFFD with 1% chokeberry powder group, HFFD+J: HFFD with 1% jujube fruits powder group, HFFD+M: HFFD with 0.5% chokeberry + 0.5% jujube fruits mixed powders group

### Organ weight and organ coefficient

Based on the results of organ weight (Table 4) and coefficient of each organ, a significant difference was observed in liver, abdominal, and epididymal fat (*p* < .0001 for each group). The weight of liver, abdominal, and epididymal fat in the HFFD group increased compared with those of the ND group. The liver weight of the HFFD+C, HFFD+J, and HFFD+M groups was significantly lower than the hepatic weight of the HFFD group (*p* < .0001), and abdominal fat and epididymal fat mass decreased in the group fed with chokeberry and/or jujube (*p* < .0001, respectively). Organ coefficients were calculated using the liver, abdominal fat, epididymal fat, heart, kidney, and lung mass.

**Table 4:** Organ Weight of Each Group

Organ weight (g)	ND	HFFD	HFFD+C	HFFD+J	HFFD+M
Liver	0.90 ± 0.09^c^	1.18 ± 0.10^a^	1.04 ± 0.12^b^	0.98 ± 0.08^bc^	0.93 ± 0.09^bc^
Abdominal fat	0.16 ± 0.07^d^	0.81 ± 0.15^a^	0.32 ± 0.13^c^	0.55 ± 0.18^b^	0.46 ± 0.16^bc^
Epididymal fat	0.79 ± 0.14^d^	2.46 ± 0.47^a^	1.14 ± 0.24^cd^	1.58 ± 0.40^b^	1.39 ± 0.41^bc^
Heart	0.13 ± 0.01^c^	0.15 ± 0.02^a^	0.14 ± 0.01^ab^	0.13 ± 0.01^c^	0.13 ± 0.01^bc^
Kidney	0.33 ± 0.03^ns^	0.36 ± 0.03	0.38 ± 0.04	0.33 ± 0.03	0.33 ± 0.04
Lung	0.17 ± 0.02^ns^	0.20 ± 0.02	0.20 ± 0.05	0.18 ± 0.02	0.18 ± 0.01

Values are means ± SD (n = 7). The different letters (a, b, c, d) within a column indicate significant difference (*p* < .05) determined by Duncan multiple range test. Abbreviation: ns, not significant. ND: normal diet group, HFFD: high fat and high fructose (HFFD) diet group, HFFD+C: HFFD with 1% chokeberry powder group, HFFD+J: HFFD with 1% jujube fruits powder group, HFFD+M: HFFD with 0.5% chokeberry + 0.5% jujube fruits mixed powders group

### Serum lipid profiles

**Fig. 3:**
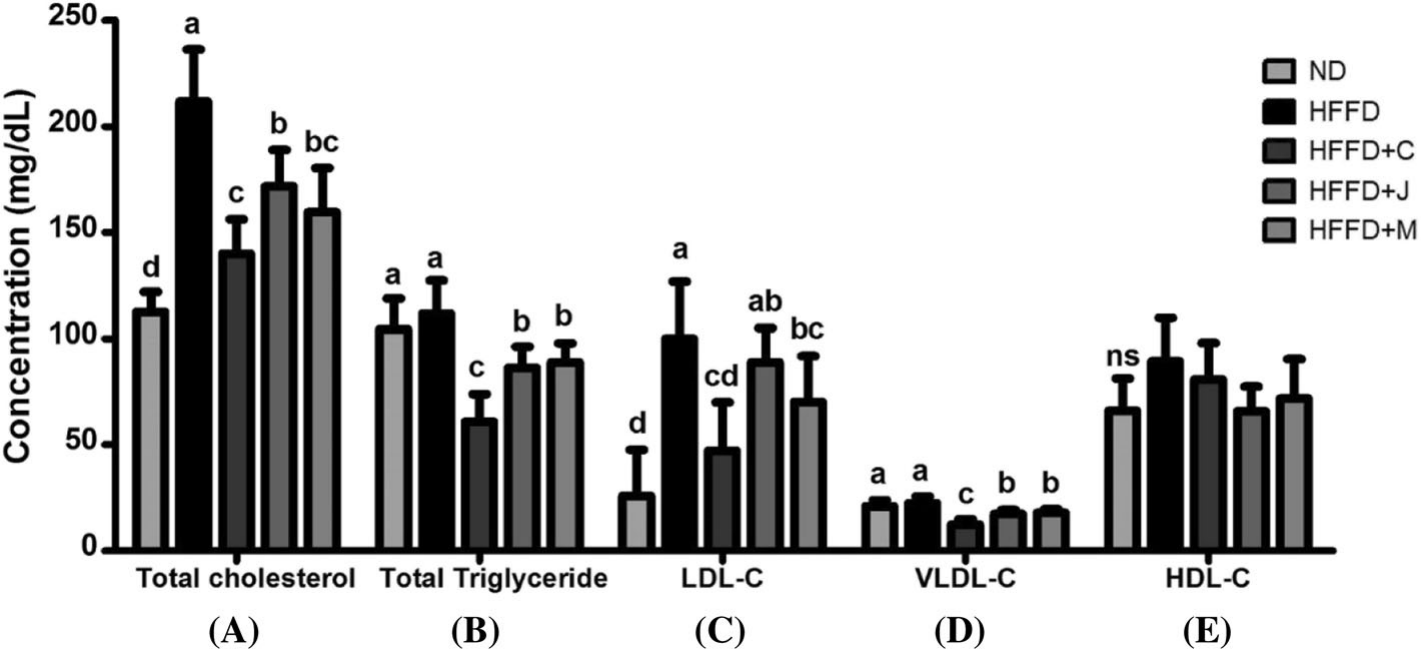
Serum Lipid Profiles. (**a**) Total cholesterols, TC; (**b**) Total triglyceride, TG; (**c**) LDL-cholesterols, LDL-C; (**d**) VLDL-cholesterols, VLDL-C; (**e**) HDL-cholesterols, HDL-C; Values are means ± SD (*n* = 7). The different letters (a, b, c, d) within a column indicate significant difference (*p* < .05) determined by Duncan multiple range test. Abbreviation: ns, not significant. ND: normal diet group, HFFD: high fat and high fructose (HFFD) diet group, HFFD+C: HFFD with 1% chokeberry powder group, HFFD+J: HFFD with 1% jujube fruits powder group, HFFD+M: HFFD with 0.5% chokeberry + 0.5% jujube fruits mixed powders group

The serum lipid profiles are shown in Fig. 3. The administration of HFFD significantly induced higher levels of serum TG, TC, LDL-C, and VLDL-C levels (*p* < .0001 for all). However, serum HDL-C levels were not affected (*p* = 0.0614). After 10 weeks of administration, the HFFD+C, HFFD+J, and HFFD+M groups had significantly lower serum TG, TC, and VLDL-C levels relative to those level of HFFD group. The LDL-C levels (Fig. 2 (C)) of the HFFD+C and HFFD+M groups significantly differed from those of the HFFD group. Serum HDL-C levels, as shown in Fig. 2 (E), were not significantly different in all groups; however, a decreasing trend was observed in the HFFD+C, HFFD+J, and HFFD+M groups compared with the HFFD group.

### Serum glucose profiles and OGTTs

**Fig. 4:**
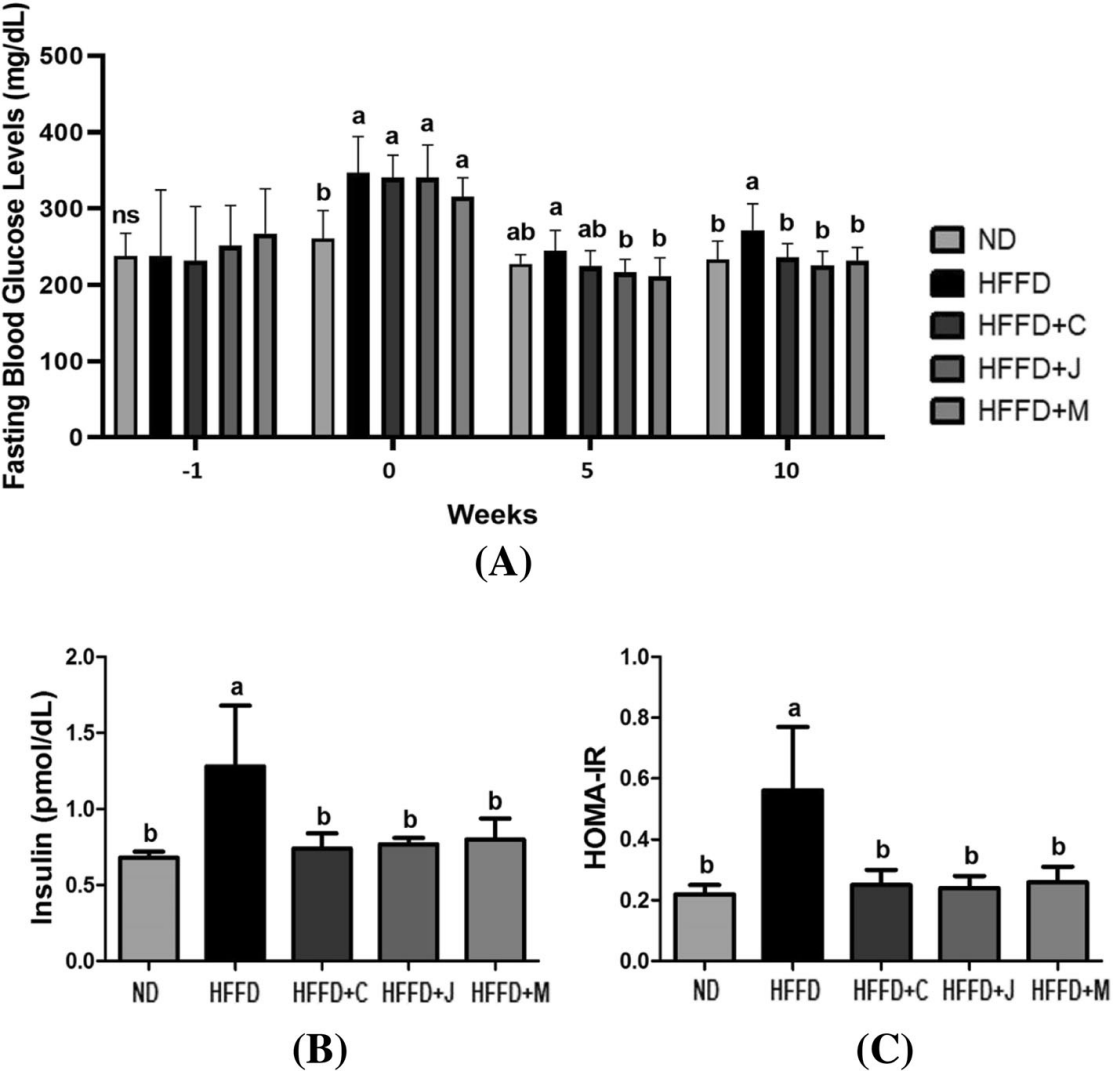
Serum Glucose Profiles. **a** Fasting blood glucose of each week; **b** Serum insulin; (**c**) HOMA-IR; Values are means ± SD (*n* = 7). The different letters (a, b) within a column indicate significant difference (*p* < .05) determined by Duncan multiple range test. Abbreviation: ns, not significant. ND: normal diet group, HFFD: high fat and high fructose (HFFD) diet group, HFFD+C: HFFD with 1% chokeberry powder group, HFFD+J: HFFD with 1% jujube fruits powder group, HFFD+M: HFFD with 0.5% chokeberry + 0.5% jujube fruits mixed powders group

Figure 4 shows the serum glucose profiles, fasting serum glucose levels, index of HOMA-IR, and serum insulin levels. The HOMA-IR score was calculated with fasting serum glucose and insulin levels. Fasting serum glucose levels of the HFFD+C, HFFD+J, and HFFD+M groups were significantly lower than that of the HFFD group (*p* = 0.0066). Both HOMA-IR score and serum insulin level were significantly lower in the HFFD+C, HFFD+J, and HFFD+M groups than in the HFFD group (*p* < .0001).

**Fig. 5:**
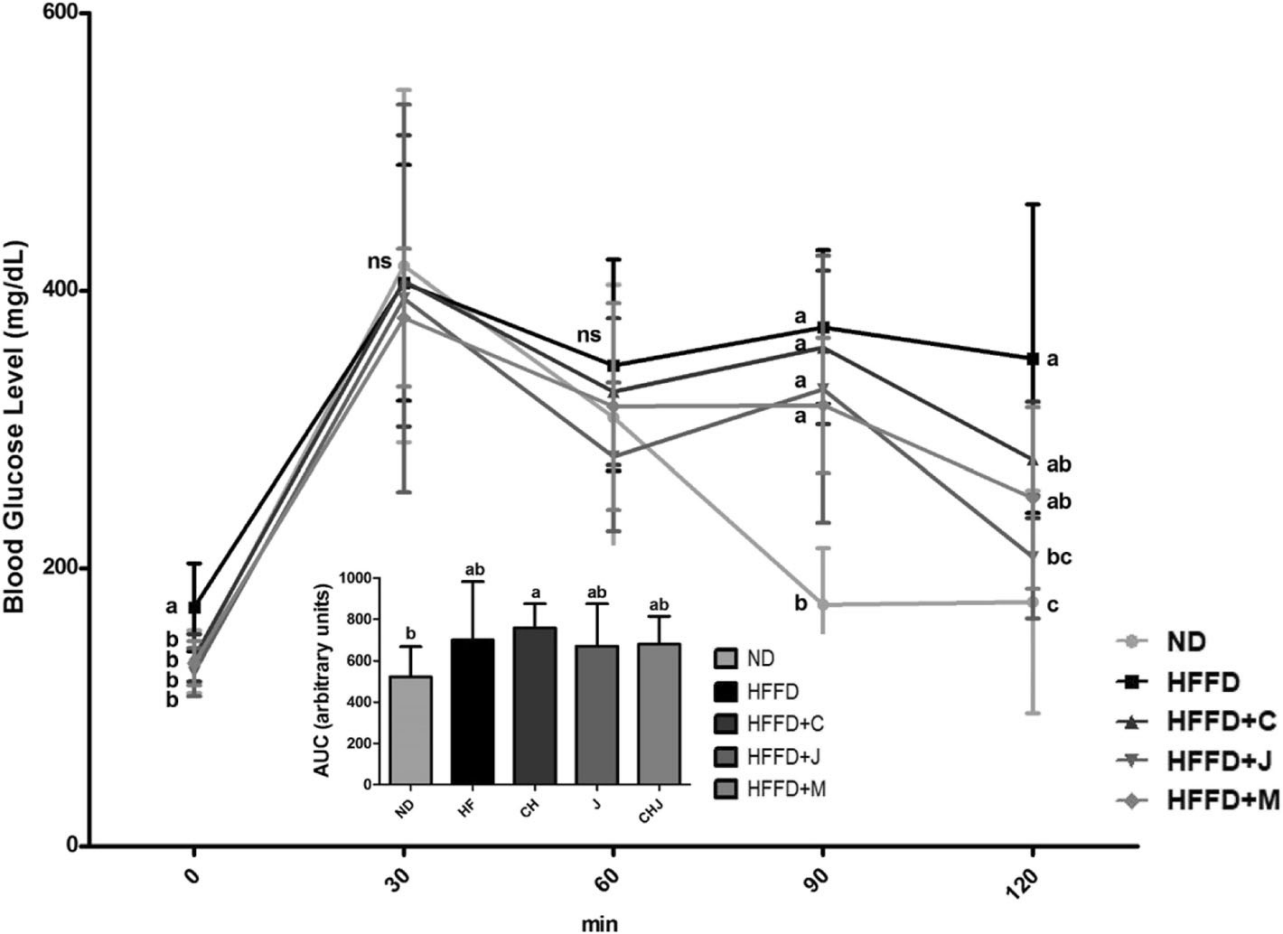
Oral Glucose Tolerance Tests at 0, 30, 60, 90, and 120 min. Values are means ± SD (*n* = 7). The different letters (a, b) within a column indicate significant difference (p < .05) determined by Duncan multiple range test. Abbreviation: ns, not significant. ND: normal diet group, HFFD: high fat and high fructose (HFFD) diet group, HFFD+C: HFFD with 1% chokeberry powder group, HFFD+J: HFFD with 1% jujube fruits powder group, HFFD+M: HFFD with 0.5% chokeberry + 0.5% jujube fruits mixed powders group

OGTTs were carried out after 9 weeks. The test results are shown in Fig. 5. At 0 min, the fasting blood glucose level of the HFFD group was significantly higher than that of the four groups (*p* = 0.0066). After the administration of 2 g/kg of D-glucose solution, no significant difference was observed at 30 and 60 min in all groups. The HFFD groups had the highest fasting blood glucose value among all groups after 120 min of glucose administration. The HFFD+C and HFFD+M groups had decreased trends at 120 min compared with the HFFD group; however, the difference was not statistically significant. The HFFD+J group had a significantly lower blood glucose level after 2 h of glucose administration than the HFFD group (*p* = 0.0029). The HFFD group showed a higher area under the curve (AUC) than the ND group. However, the difference was not significant.

### AI and CRF

**Fig. 6:**
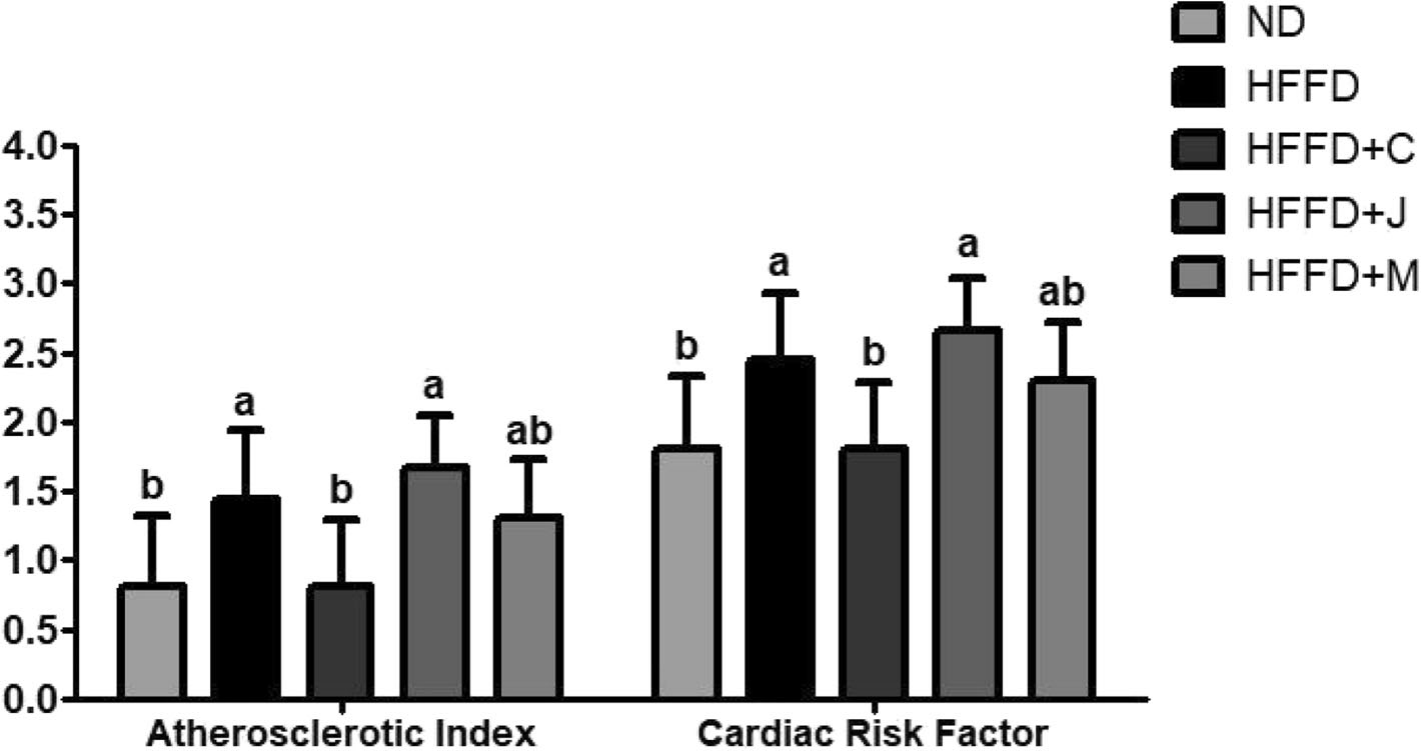
Atherosclerotic Index and Cardiac Risk Factor of Each Group. Values are means ± SD (*n* = 7). The different letters (a, b) within a column indicate significant difference (p < .05) determined by Duncan multiple range test. Abbreviation: ND: normal diet group, HFFD: high fat and high fructose (HFFD) diet group, HFFD+C: HFFD with 1% chokeberry powder group, HFFD+J: HFFD with 1% jujube fruits powder group, HFFD+M: HFFD with 0.5% chokeberry + 0.5% jujube fruits mixed powders group

AI and CRF were calculated using the Haglund method [37], and the results were as follows: [1] AI: ND (0.80 ± 0.48^b^), HFFD (1.45 ± 0.45^a^), HFFD+C (0.81 ± 0.44^b^), HFFD+J (1.66 ± 0.35^a^), and HFFD+M (1.30 ± 0.39^ab^) (*p* = 0.00340) and [2] CRF: ND (1.80 ± 0.48^b^), HFFD (2.45 ± 0.45^a^), HFFD+C (1.81 ± 0.44^b^), HFFD+J (2.66 ± 0.35^a^), and HFFD+M (2.30 ± 0.39^ab^) (*p* = 0.00340). (Fig. 6).

### Liver TG

**Fig. 7:**
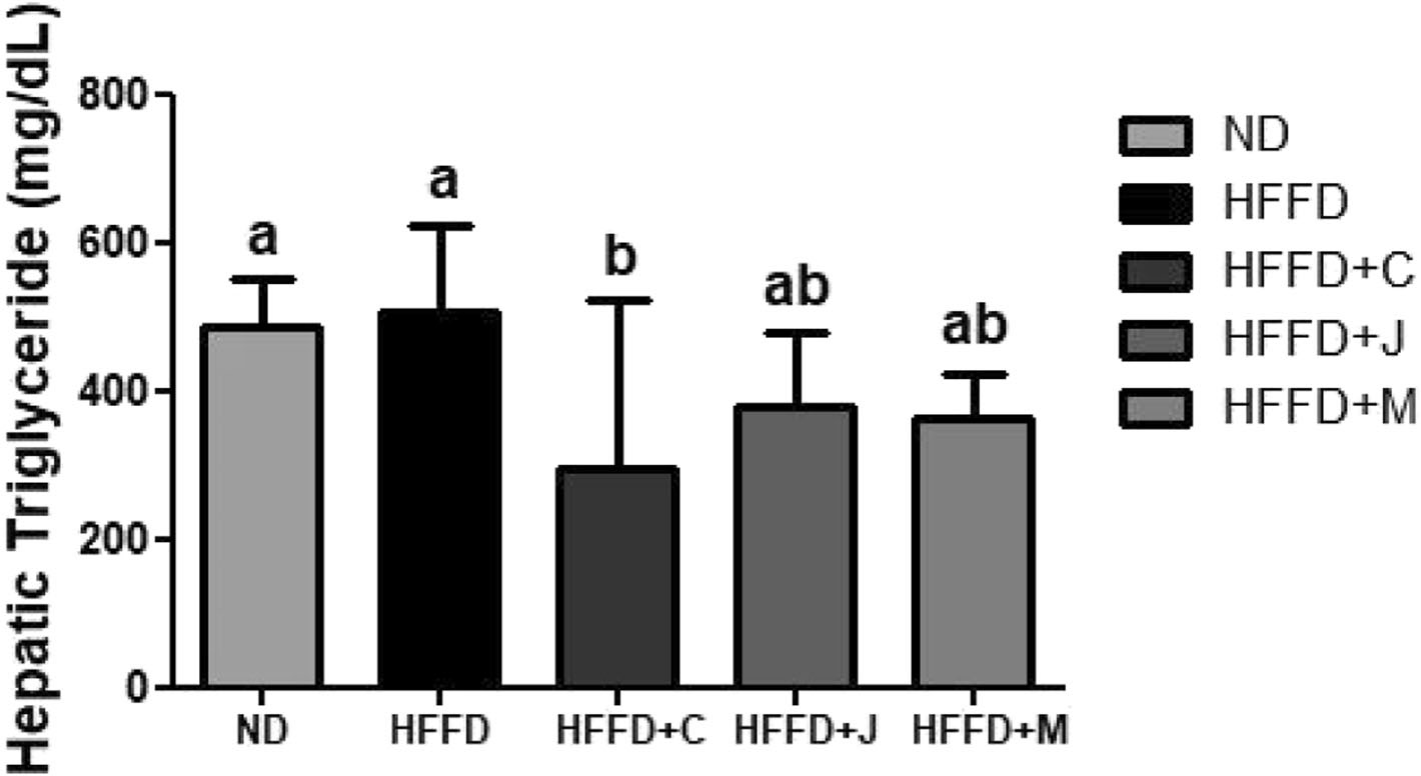
Liver Triglycerides of Each Group. Values are means ± SD (*n* = 7). The different letters (a, b) within a column indicate significant difference (p < .05) determined by Duncan multiple range test. ND: normal diet group, HFFD: high fat and high fructose (HFFD) diet group, HFFD+C: HFFD with 1% chokeberry powder group, HFFD+J: HFFD with 1% jujube fruits powder group, HFFD+M: HFFD with 0.5% chokeberry + 0.5% jujube fruits mixed powders group

The TG levels of the mice are presented in Fig. 7 and Table 5. That of the HFFD group was 508.67 ± 105.07 mg/dL, and it was the highest among all groups. The liver TG values of the HFFD+J and HFFD+M groups were not significantly compared with those of the HFFD group. Moreover, a decreasing trend was observed. The TG level of the HFFD+C group (293.33 ± 211.14 mg/dL) was significantly lower than the HFFD group (*p* = 0.0192).

**Table 5:** Liver Triglycerides of Each Group

	Liver TG (mg/dL)
ND	488.57 ± 57.01^a^
HFFD	508.67 ± 105.07^a^
HFFD+C	293.33 ± 211.14^b^
HFFD+J	380.67 ± 91.26^ab^
HFFD+M	363.90 ± 54.89^ab^

Values are means ± SD (n = 7). The different letters (a, b) within a column indicate significant difference (p < .05) determined by Duncan multiple range test. Abbreviation: ns, not significant. ND: normal diet group, HFFD: high fat and high fructose (HFFD) diet group, HFFD+C: HFFD with 1% chokeberry powder group, HFFD+J: HFFD with 1% jujube fruits powder group, HFFD+M: HFFD with 0.5% chokeberry + 0.5% jujube fruits mixed powders group

### Hepatic protein expression of IR, IRS-1, p-PI3K, PI3K, p-Akt, Akt, and CAT

**Fig. 8:**
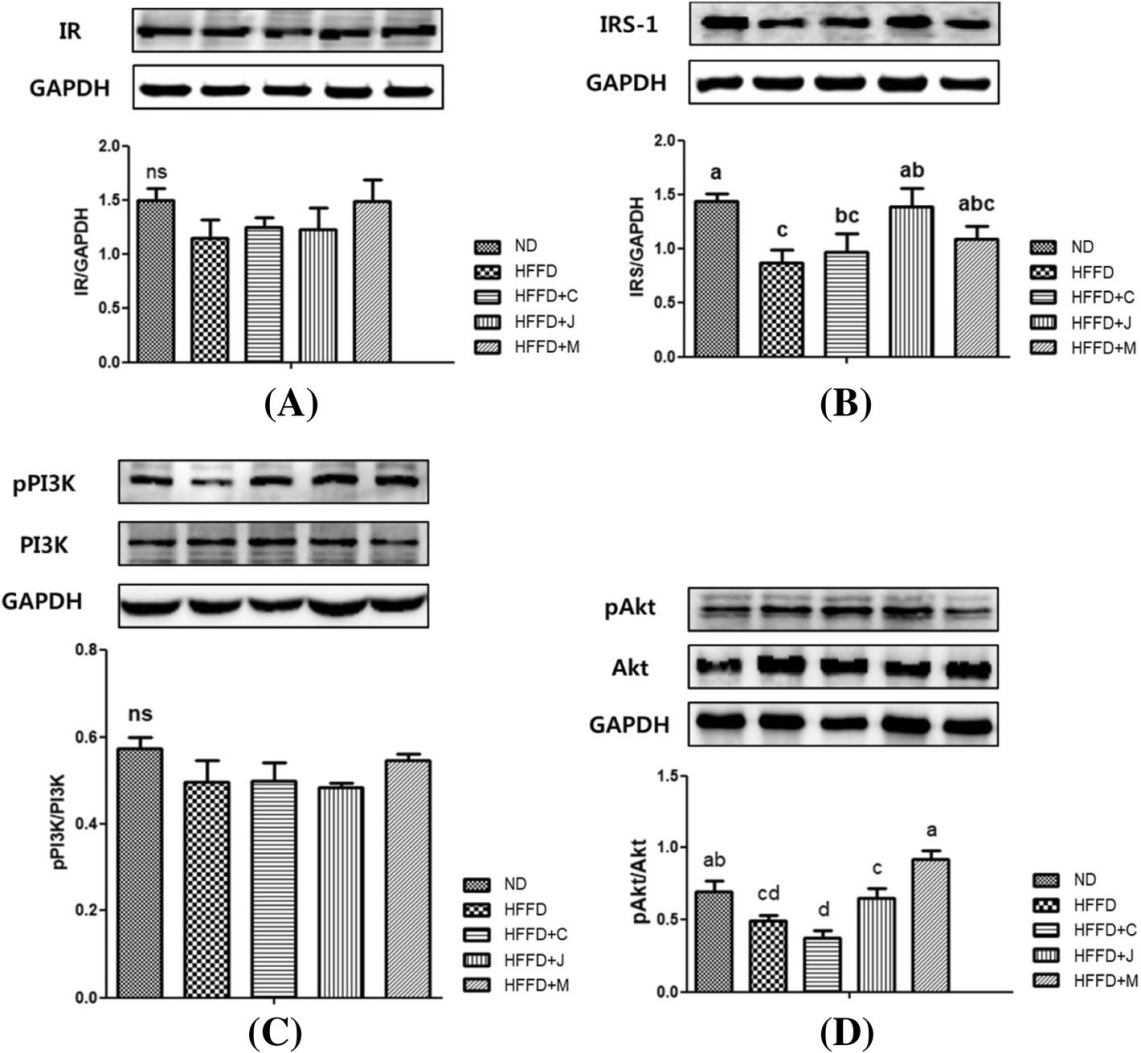
Hepatic Protein Expression of IRS-1/PI3K/Akt Pathway of Each Group. (**a**) IR; (**b**) IRS-1; (**c**) p-PI3K/PI3K; (**d**) p-Akt/Akt; Values are means ± SD (*n*= 7 ). The different letters (a, b) within a column indicate significant difference (p < .05) determined by Duncan multiple range test. Abbreviation: ns, not significant. ND: normal diet group, HFFD: high fat and high fructose (HFFD) diet group, HFFD+C: HFFD with 1% chokeberry powder group, HFFD+J: HFFD with 1% jujube fruits powder group, HFFD+M: HFFD with 0.5% chokeberry + 0.5% jujube fruits mixed powders group

**Fig. 9:**
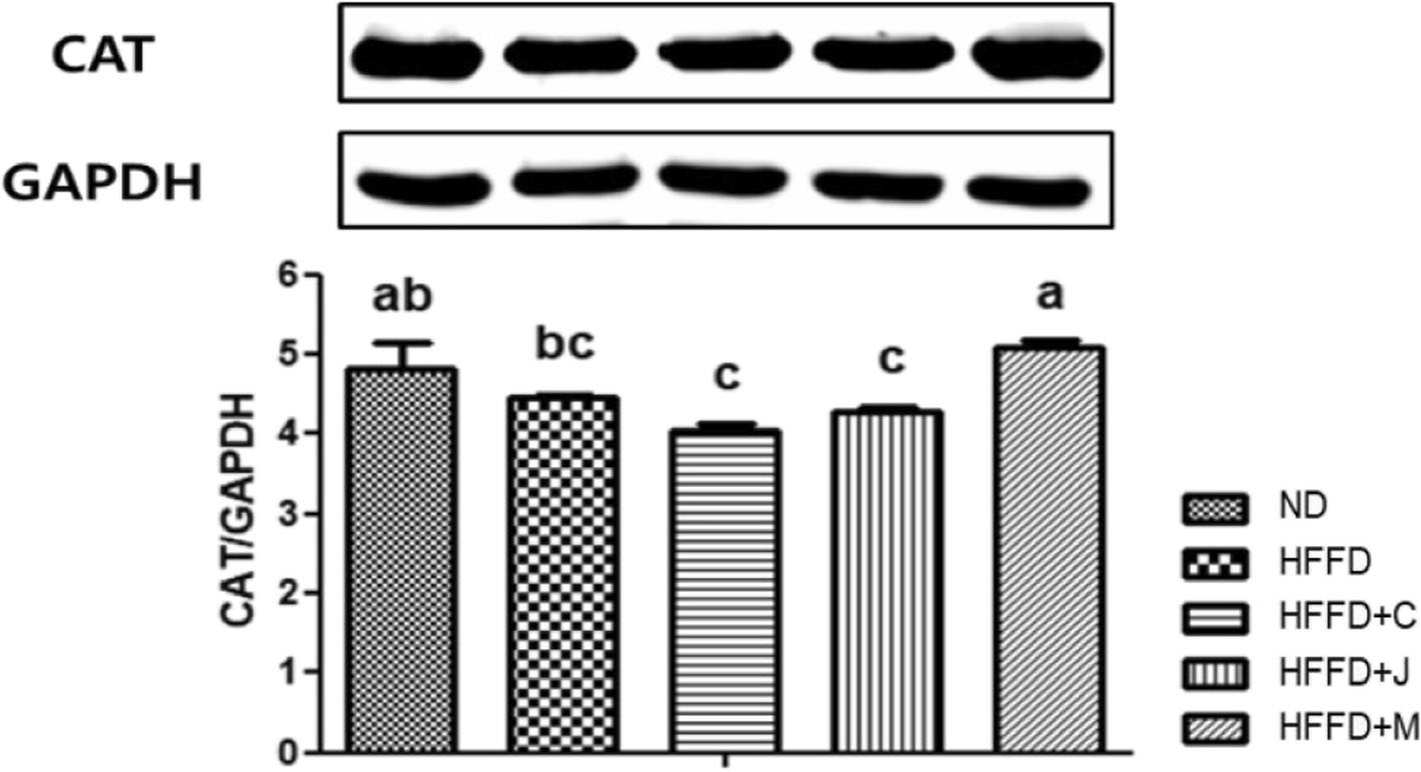
Hepatic Protein Expression of CAT of Each Group. Values are means ± SD (*n* = 7). The different letters (a, b) within a column indicate significant difference (*p* < .05). Abbreviation: ns, not significant. ND: normal diet group, HFFD: high fat and high fructose (HFFD) diet group, HFFD+C: HFFD with 1% chokeberry powder group, HFFD+J: HFFD with 1% jujube fruits powder group, HFFD+M: HFFD with 0.5% chokeberry + 0.5% jujube fruits mixed powders group

Results of the representative Western blot analysis of IR, IRS-1, PI3K, p-PI3K, Akt, p-Akt, and CAT are shown in Figs. 8 and 9.

IR is acted by insulin, which is secreted by controlling glucose/lipid metabolism. These factors increase insulin secretion, and insulin is then activated by IR tyrosine kinase, resulting to phosphorylated and activated insulin receptor substrate 1 (IRS-1). The IR expressions were lower in the four HFFD-fed groups than the ND group. However, the difference was not statistically significant among all five groups. The hepatic IRS-1 expression showed a compelling change in the difference between the groups. The expression in the HFFD+C and HFFD+M groups increased although the result was not statistically significant. Meanwhile, the increase in the expression in the HFFD+J group was more significant than that of the HFFD group (*p <* .05).

The PI3K/Akt pathway was analyzed via western blot analysis to monitor the activation of PI3K/Akt proteins by consumption of chokeberry and/or jujube on HFFD-induced metabolic disorders. The phosphorylation level of PI3K and Akt was calculated using the p-PI3K/PI3K and p-Akt/Akt ratio.

The phosphorylated PI3K/PI3K in the liver did not significantly differ among the five groups. A decreasing trend was observed among the HFFD, HFFD+C, HFFD+J, and HFFD+M groups compared with the ND group. Although it was not statistically significant, in terms of phosphorylated PI3K/PI3K, an increasing trend was observed in the HFFD+M group compared to the other groups.

Akt is downstream to PI3K, and it can be phosphorylated and activated via PI3K activation. In Fig. 8, the pAkt-to-Akt ratio of the HFFD groups was significantly lower than that of the ND groups (*p* = 0.003). A significantly higher expression was observed in the HFFD+M group than in the HFFD control group (*p* = 0.003).

Figure 9 shows that hepatic protein expression of CAT, and results were statistically significant among the five groups. CAT is considered an important enzyme because it protects the cell from oxidative stress by reactive oxygen species (ROS). The HFFD+M group had a significantly higher expression of CAT than the HFFD group, and this value was comparable to that of the ND group.

## Discussion

Daily consumption of fruits can improve various biomarkers related to obesity because of their antioxidant properties. Previous studies have reported that chokeberry and jujube fruits have antioxidative properties because they contain polyphenols and flavonoids [20, 29, 30, 39, 40]. We previously measured the total phenol, flavonoids, and DPPH radical scavenging activity. The results of them were as follows: total phenols levels (chokeberry, 968.47 mg GAE/100 g; jujube fruits, 518.65 mg GAE/100 g; mixture of chokeberry and jujube fruit, 521.81 mg GAE/100 g), flavonoid levels (chokeberry, 374.08 mg QE/100 g; jujube fruits, 199.92 mg QE/100 g; mixture of chokeberry and jujube fruit, 244.08 1 mg QE/100 g), and DPPH radical scavenging activity (chokeberry, 78.59%; jujube fruit, 85.30%; mixture of chokeberry and jujube fruit, 82.32%. all of them compared with Ascorbic acid, 92.59%). We administered the same chokeberry and jujube powder to animals used in the current study. Therefore, this current study investigated the protective effects of chokeberry and/or jujube on lipid and glucose metabolism by attenuating the metabolic alteration associated with HFFD in C57BL/6 J mice. The high fat and high fructose diet-induced MetS mouse model has been used as a valuable rodent model [41, 42]. In previous studies, most HFFDs contained 45–60% of fat and up to 30% of fructose with drinking water [41–48]. Results showed that HFFD increased body weight and abdominal fat mass and reduced glucose function with insulin sensitivity [42]. Moreover, HFFD was correlated to insulin resistance and impaired lipid metabolism [48, 49].

To the best of our knowledge, this study first investigated the effects of dietary chokeberry and/or jujube fruits in mice models with HFFD-induced metabolic disorder. The present study confirmed that HFFD induced obesity and increased the level of body weight changes. Moreover, HFFD caused increased fat accumulation in abdominal and epididymal regions. These results demonstrated that HFFD is correlated to central obesity in mice.

The body weight of the ND and HFFD groups did not differ at the start of the study. However, differences were observed 10 weeks after the administration of HFFD. That is, the body weight of the HFFD group was 1.45-fold higher than that of the ND group. The changes in body weight of HFFD+C, HFFD+J, and HFFD+M group had persuasive decline in their weight compared to that of HFFD group. In this regard, dietary consumption of chokeberry and jujube fruits lead to lose the weight effectively in dyslipidemia and insulin resistance mice model induced by HFFD. Compared with the results of a review article [50], polyphenol rich foods have anti-obesity effects via several mechanisms involving suppression of adipocyte differentiation and proliferation, and inhibition of de novo lipogenesis and fatty acid oxidation. Our results also indicated that weight and organ weight were reduced when fruits containing polyphenols were consumed.

Based on our results, calorie intake per day was not different among all groups. Our results showed that the increase in body weight was not attributed to an increase in energy intake. The FER (%) increased the value of the HFFD group than the ND group. That is, the mice fed with HFFD gained more weight per equal feed intakes than mice fed with normal diet. Chokeberry and/or jujube suppressed FER (%) in this study.

In mice fed with HFFD, the higher levels of lipid profiles in the serum were associated with dyslipidemia [48, 51]. The present study showed increased serum TG, TC, HDL-C, non HDL-C, LDL-C, and VLDL-C levels, which is in accordance with previous studies. Chokeberry and/or jujube fruit diet had beneficial effects on attenuating serum lipid profiles but not HDL-C levels. Previous studies have shown that polyphenol-rich products can decrease serum lipid parameters leading to relieve the risk of dyslipidemia [6, 52–54]. Our findings indicated that dietary chokeberry and/or jujube have protective effects against dyslipidemia.

Both fasting blood glucose and serum insulin levels are important in HFFD-induced mice model. HFFD feeding is an important dietary pattern associated with abdominal adiposity and insulin resistance [55]. High caloric diet with fructose-enriched drinks is a proper method for inducing hepatic insulin resistance in rodent models [43]. This diet is a major contributor to insulin resistance because rodents that were fed with such diet had a higher HOMA-IR score. Shuang Mei et al. [56] have shown that a small amount of dietary carbohydrate can promote HFFD-induced insulin resistance to induce maximal levels. This study showed that HFFD increased fasting glucose concentration, HOMA-IR score, and serum insulin level in the HFFD group compared with the ND group. That is, HFFD feeding causes higher insulin resistance, hyperinsulinemia, and hyperglycemia. Chokeberry and/or dried jujube fruit diet significantly lowers the levels of fasting serum glucose and insulin and HOMA-IR score. Our findings indicated that the consumption of chokeberry and jujube fruit with HFFD might lower serum glucose profiles associated with insulin resistance due to their rich polyphenol contents.

OGTTs are performed to examine the effect of dietary chokeberry and/or dried jujube fruits on insulin sensitivity and glucose homeostasis. High fat and high carbohydrate (sucrose) treatment in mice resulted in increased plasma insulin level and units of AUC during OGTTs compared with normal chow diet [6]. In this study, the HFFD group had the highest point among the five groups after 120 min of D-glucose solution administration. In addition, the consumption of chokeberry and/or dried jujube fruits lowered blood glucose levels. In relation to this, our results showed that dietary chokeberry and/or dried jujube fruits may have protective effects against HFFD-related hyperglycemia and insulin sensitivity.

In a review article [57], insulin signaling is associated with the regulation of glucose and lipid metabolism by activating the downstream, such as the PI3K/Akt pathway. After secreted insulin combines with insulin receptor, which is located in the cell membrane, it activates IRS-1 and IRS-2, leading to the subsequent activation of the PI3K/Akt pathway. The aforementioned signaling cascade positively regulated cell growth, metabolism, and survival [12]. The results of the current study are similar to those of a review paper showing that the loss of IRS-1 is indicative of insulin resistance, and this loss is correlated to the inactivation of hepatic PI3K and Akt resulting in diabetes (hyperglycemia and hyperinsulinemia) and hypolipidemia [12]. Incidentally, the HFFD+C, HFFD+J, and HFFD+M groups showed an increasing trend compared with the HFFD group. In particular, the IRS-1 expression of HFFD+J group was significantly higher than the HFFD group. Therefore, the intake of fruits reduces the severity of insulin resistance via induction of IRS-1 expression in the liver.

PI3K interacts with Akt mainly in insulin signaling. The serine/threonine kinase Akt, which is also called protein kinase B (PKB), is an important protein in cell signaling downstream of insulin stimuli due to glucose transporter 4 (GLUT4) [18]. Studies that examined Akt expression in models with insulin resistance have shown increased Akt phosphorylation compared with normal groups [58, 59]. Another study has revealed reduction of p-Akt/Akt ratio in the HFFD group compared with the normal diet group [60]. According to an Akt article, Akt inactivation resulted in the impairment of systemic glucose and lipid homeostasis and body weight control in the development of MetS [12]. In relation to this, our study results about Akt expression in the liver were similar to those of a previous study. The HFFD+J and HFFD+M groups had activated expression of p-Akt/Akt in liver compared with HFFD group, but the expression of HFFD+M group only showed a convincing increase in comparison with HFFD group. In summary, the IRS-1 and p-Akt/Akt expressions decreased in the HFFD group and increased in the fruit-fed group, it suggested that the intake of the fruits can reduce the risk of developing MetS in mice fed with HFFD. The molecular mechanisms by which flavonoids and phenolic acids stimulate the expression of proteins involving IRS/PI3K/Akt pathway were reported by previous studies [10, 19, 59, 61–63]. Activation of IRS/PI3K/Akt pathway promotes the downstream markers such as FoxO1, GSK-3, mTORC1, and SREBP, which are major regulators of glucose and lipid metabolism. Our results indicated that chokeberry and jujube fruit may have therapeutic potency for alleviating the risk of MetS.

CAT is associated with obesity or insulin resistance, and it is a decomposing enzyme from H_2_O_2_ to O_2_ and H_2_O, resulting in oxidative stress plays an important role in the development of MetS [64]. In a review article [65], CAT polymorphisms were associated with the risk of developing diabetes mellitus since CAT decreased due to other factors such as genetics and environments. In our results, a valid value was only observed in the HFFD+M group. It indicated that mixed fruit feeding along with western-style HFFD diet may reduce the severity of insulin resistance. Since S. Wang et al. [66] have shown that the combination of different foods could have synergistic effects on total antioxidant capacities because mixed foods have more various bioactive compounds than single food. Taken together, our results would have been relatively better in the HFFD+M group. According to the previous study [67], activation of CAT is main to the induction of cellular antioxidant system, which can improve insulin resistance in in-vivo study [68]. They also confirmed that expression of inflammatory factors such as CAT by HFD-induced via PI3K/AKT/JNK pathway [67].

In summary, the current study investigated to elucidate the effect of individual or combined fruits consumption on dyslipidemia and insulin resistance-induced mice by high fat and high fructose diet. By daily intake of fruits for 10 weeks, there were significant reduction in body weight, organ weight, serum lipid profiles, serum glucose profiles, leading to upregulate hepatic protein expression of IRS/PI3K/Akt and CAT protein expression. Our results might be suggested that daily consumption of fruits has the potential to provide therapeutic help to alleviate dyslipidemia and insulin resistance by HFFD diet. In addition, this study showed that consumption of chokeberry with jujube has not only better taste but also more metabolic benefits than eating alone. The development of functional food with chokeberry, as known as superfood, has been attempted in South Korea. Our research provides scientific evidences and proposes as a potential therapeutic agent for functional foods. Based on the results of the present study, commercial availability for functional food development might be provided. There are limitations in this study due to lack of downstream effectors on the IRS/PI3K/Akt pathway. Therefore, direct or indirect findings will need to be developed through further studies.

## Conclusion

**Fig. 10:**
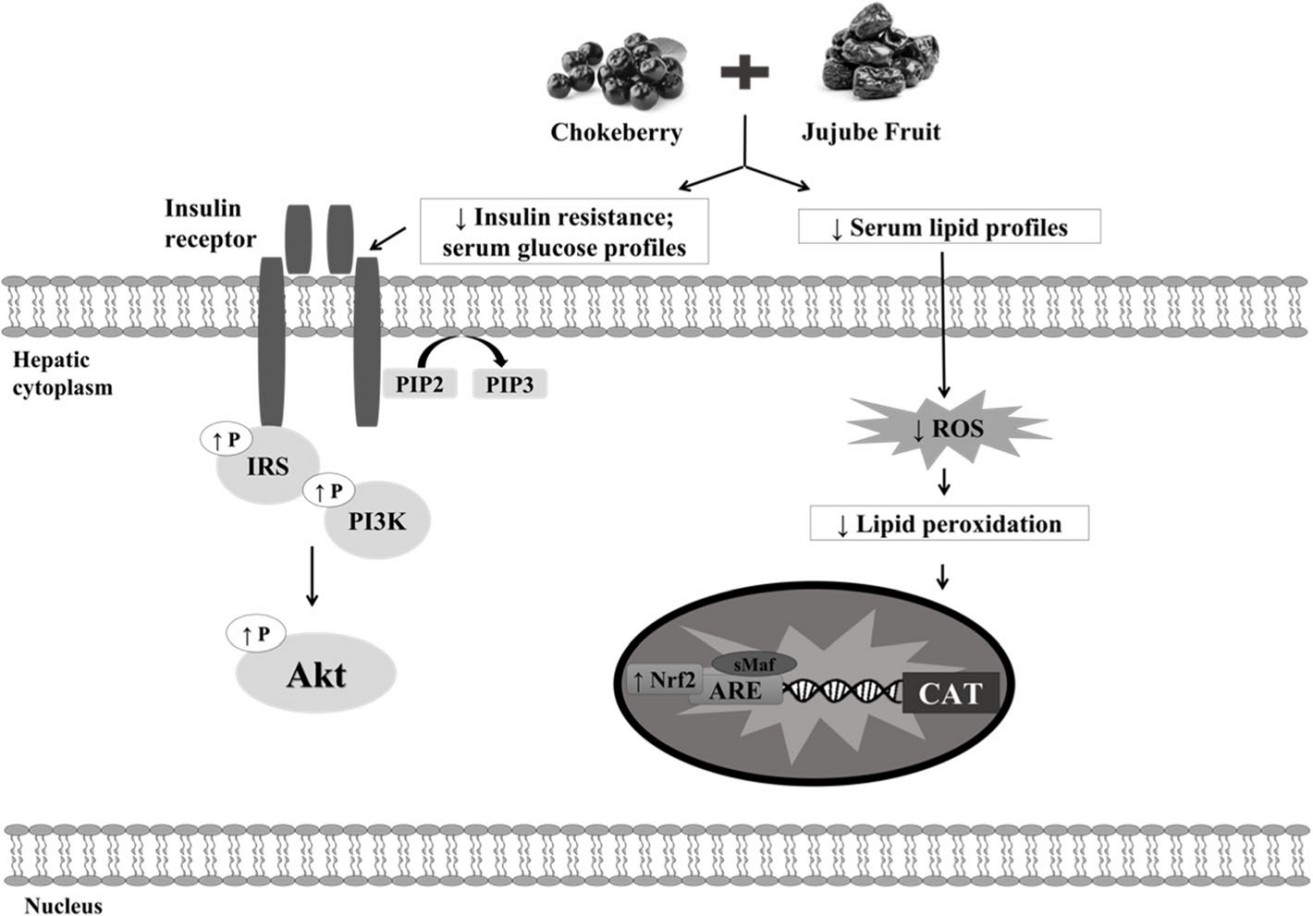
The Proposed Mechanism for Chokeberry and Jujube’s action in liver. Chokeberry and jujube activate IRS/PI3K/Akt pathways on dyslipidemia and insulin resistance mice induced HFFD diet

The present study showed that the HFFD diet caused obesity, insulin resistance, hyperinsulinemia, and dyslipidemia associated with MetS through impaired parameters such as increased body weight, cholesterol levels (TC, TG, LDL, VLDL, and non HDL), fasting glucose concentration, and insulin level. The consumption of dietary chokeberry and dried jujube fruits improved the aforementioned indicators in this study. Dietary chokeberry and dried jujube fruits had protective effects in HFFD-fed mice via activation of IRS-1, p-Akt/Akt, and CAT in the liver. Hence, based on these results, the combination of chokeberry and jujube fruits may have therapeutic effects against obesity, insulin resistance, and MetS (Fig. 10).

## Abbreviations

AI: Atherosclerotic index
CAT: Catalase
CRF: Cardiac risk factor
FER: Food efficiency ratio
HDL-C: High-density lipoprotein cholesterol
HFFD: High fat and high fructose diet
HFFD+C: HFFD with chokeberry powder
HFFD+J: HFFD with dried jujube fruit powder
HFFD+M: HFFD with chokeberry and dried jujube fruit mixed powder
HOMA-IR: Homeostatic model assessment-insulin resistance
IR: Insulin receptor
IRS-1: Insulin receptor substrate 1
LDL-C: Low-density lipoprotein cholesterol
MetS: Metabolic syndrome
OGTTs: Oral glucose tolerance tests
PI3K: Phosphoinositide 3-kinase
PVDF: Polyvinylidene difluoride
TC: Total cholesterol
TG: Total triglyceride
VLDL-C: Very-low-density lipoprotein cholesterol
